# Hypocholesterolemic effect of β-caryophyllene in rats fed cholesterol and fat enriched diet

**DOI:** 10.3164/jcbn.17-3

**Published:** 2018-02-07

**Authors:** Amani A. Harb, Yasser K. Bustanji, Shtaywy S. Abdalla

**Affiliations:** 1 Department of Biological Sciences, Faculty of Science, The University of Jordan, Amman 11942, Jordan; 2 Faculty of Pharmacy, The University of Jordan, Amman 11942, Jordan

**Keywords:** antioxidant, β-caryophyllene, fatty liver, hypercholesterolemia, lipids

## Abstract

Hypercholesterolemia is a major risk factor for cardiovascular diseases. This study investigated the cholesterol-lowering potential of β-caryophyllene in a rat model. Hypercholesterolemia was induced by feeding male Wistar rats a high cholesterol and fat diet for 2 weeks. This was followed by oral administration of β-caryophyllene to hypercholesterolemic rats at 30, 100 and 300 mg/kg b.w. for 4 weeks. A dose of 30 mg/kg of β-caryophyllene significantly lowered serum total cholesterol, low density lipoprotein and the atherogenic index and significantly increased high density lipoprotein level. Moreover, it ameliorated liver injury as evidenced by decreasing hepatomegaly, macrovesicular steatosis and the activity of hepatic marker enzymes alanine aminotransferase and aspartate aminotransferase. Furthermore, it increased the activity of the antioxidant enzyme superoxide dismutase. This dose of β-caryophyllene significantly inhibited the activity of hepatic hydroxy-methylglutaryl coenzyme A reductase. Higher doses (100 and 300 mg/kg) of β-caryophyllene, however, did not induce significant beneficial effects on the studied parameters. These observations demonstrate that β-caryophyllene has a cholesterol-lowering effect on hypercholesterolemic rats, thus offering protection against hypercholesterolemia-induced diseases such as atherosclerosis and fatty liver.

## Introduction

Hyperlipidemia, particularly hypercholesterolemia, is known to be a major contributor to the development of cardiovascular disease, fatty liver disease and carcinogenesis leading to health problems and death around the world.^(1)^ A diet containing a large amount of fat was found to be associated with increased risk of obesity and hyperlipidemia in human and rodents as it increases cholesterol and triglyceride (TG) levels in plasma and tissues.^(2)^

Presently, pharmacological drugs to treat hyperlipidemia effectively are available but these have a myriad of adverse effects, a matter that has motivated researchers to seek natural alternatives.^(3)^ For example, natural antioxidant compounds have been shown to be useful in attenuating the extent of hypercholesterolemia as they correct the imbalance between free radicals (pro-oxidants) resulting from increased cholesterol level and anti-free radicals (antioxidants).^(4,5)^

The dietary sesquiterpene β-caryophyllene (BCP) is a volatile compound found as a major component of the essential oils of many food and spice plants, including black pepper (*Piper nigrum*),^(6)^ oregano (*Oreganum vulgare*),^(7)^ cinnamon (*Cinnamon* spp.),^(8)^ clove (*Eugenia caryophyllata*),^(9)^ thyme (*Salvia* spp.),^(10)^ rosemary (*Rosmarinus officinalis*),^(11)^ and hops (*Humulus lupulus*) and probably many other species.^(12)^ For example, BCP was found to form up to 35% of the essential oil of the hemp plant *Cannabis sativa*.^(13)^ In addition to its uses as food additive and in cosmetics, BCP has appreciable anti-oxidant and anti-inflammatory activities, and has been used in diseases associated with inflammation and oxidative stress.^(14)^ Gertsch *et al.*^(15)^ have shown that BCP binds selectively to tetrahydrocannabinol binding site (CP55940 binding site) of the cannabinoid-2 (CB2) receptor, leading to cellular activation and anti-inflammatory effects. CB2 receptors mediate many physiological and pathological effects including inflammation, edema formation, analgesic effects, ischemia reperfusion injury, atherosclerosis and osteoporosis.^(15)^

Several observations from literature suggested that BCP may have a hypolipidemic action. First, BCP has an antioxidant activity leading to reduction of reactive oxygen species (ROS) due to its free radical-scavenging effect against hydroxyl anions, superoxide anions and lipid peroxides.^(16,17)^ Increased ROS was found to be associated with full activation of hydroxyl-methylglutaryl coenzyme A (HMG-CoA) reductase.^(18)^ This activation may lead to cholesterol synthesis and to hyperlipidemia. Reduction of ROS by BCP, therefore, may be associated with deactivation of HMG-CoA reductase and decreased cholesterol synthesis, leading to hypolipidemia. Second, activation of CB2 by BCP is known to attenuate inflammation.^(15)^ Since the inflammatory response is accompanied by higher expression of HMG-CoA reductase, cholesterol synthesis and lipoprotein synthesis which facilitates cell immune response,^(19)^ therefore, it is assumed that attenuation of inflammation by BCP decreases cholesterol biosynthesis, thus leading to hypocholesterolemia and to hypolipidemia. Third, it was found that a resorcinol-derivative of BCP decreases the inclusion of labeled sodium acetate into cholesterol in mice liver challenged with zymosan to induce aseptic inflammation.^(20)^ Fourth, BCP is also a ligand for the peroxisome proliferator-activated receptor (PPAR-α), a nuclear transcription factor, by direct interaction with the ligand-binding domain of PPAR-α.^(21)^ This nuclear receptor is the molecular target for hypolipidemic drugs such as the fibrates. Activation of PPAR-α regulates lipid metabolism by regulating the expression of genes involved in fatty acid β oxidation, TG metabolism, high density lipoprotein (HDL) metabolism, cholesterol metabolism and transport.^(22)^ PPAR-α activation leads to improving lipid profile through decreasing plasma levels of low density lipoprotein (LDL) and TG, and increasing plasma HDL levels.^(23)^ More recently, BCP was found to attenuate palmitate-induced lipid accumulation through AMPK signaling by activating CB2 receptor in human HepG2 hepatocytes.^(24)^ Finally, BCP is found in many spices that have hypolipidemic activity.^(9,25)^ Among the spices that have hypolipidemic effect and contain BCP in their chemical constituents are chili peppers (*Capsicum* spp.) and cloves (*Eugenia caryophyllata*).^(26,27)^ Moreover, essential oil of wormwood that contains 16.3% BCP has a hypocholesterolemic effect.^(28)^

BCP was approved by several food and flavor regulatory agencies for use as a food additive and was classified as a “generally regarded as safe” compound.^(11)^ This compound created an interest to many researchers as a lead for drug development,^(21)^ therefore, this work has been undertaken to test the hypothesis that BCP has a hypolipidemic effects in Wistar rats fed a high cholesterol and fat diet.

## Materials and Methods

### Chemicals

Cholesterol (purity 94%, Sigma-Aldrich, Japan), cholic acid (purity >98%, Sigma-Aldrich, New Zealand), and BCP (purity>98.5%, Sigma-Aldrich, Romania) were purchased from the indicated sources. Atorvastatin calcium was kindly provided by SANA Pharmaceuticals, Amman, Jordan. The enzymatic kits for quantitative assay of total cholesterol (TC), TG, HDL, aspartate aminotransferase (AST), alanine aminotransferase (ALT), alkaline phosphatase (ALP) and lactate dehydrogenase (LDH) were purchased from Biosystem, Barcelona, Spain. Commercial assay kits used to determine the activity of catalase enzyme (CAT) (Cayman’s Catalase Assay Kit, Item No. 707002) and superoxide dismutase enzyme (SOD) (Cayman’s Superoxide Dismutase Assay Kit, Item No. 706002) were purchased from Cayman Chemical Company (Ann Arbor, MI).

### Preparation of high cholesterol and fat diet (HCFD)

The HCFD is a standard rat chow supplemented with 1% cholic acid, 2% pure cholesterol powder, 20% fat (animal source) and 2% corn oil. The components were added gradually to the ground normal diet, fully mixed until homogenous, formed into a dough with the addition of distilled water (1,000 ml), rolled up using simple tools, cut into small pellets and allowed to dry at room temperature for 2–3 days. The diet was prepared weekly and was stored at 4°C until use to reduce oxidation. The HCFD was composed of: Carbohydrate 43.57%, crude protein 12.38%, crude fiber 4.73%, crude fat 3.17%, cholesterol 2%, cholic acid 1%, animal fat 20%, corn oil 2%, total ash 4.3% and moisture 6.85% whereas the normal diet contained 58.1% carbohydrates, 16.51% crude proteins, 0% animal fat, corn oil, cholesterol and cholic acid, with the other components are not significantly changed. Both types of diet were analyzed in the Feed Laboratory Analysis, Department of Animal Production, Faculty of Agriculture, The University of Jordan, Amman.

### Animals and experimental design

Animals were housed, fed and treated in accordance with the University of Jordan ethical guidelines for animal protection and experimental approval. Adult male Wistar rats (*n* = 48), weighing 150–180 g, were kept for one week under laboratory environment for acclimation then randomly divided into six groups (8 animals each) as follows:

Group 1 (normal control), rats were fed normal diet for 6 weeks. During the last 4 weeks of the study, the vehicle (corn oil) was given. In the remaining 5 groups, hypercholesterolemia was induced by feeding rats HCFD for 2 weeks. Rats that had total cholesterol levels above 200 mg/dl were considered as hypercholesterolemic and were randomly divided into 5 groups (groups 2–6):^(29)^

Group 2 (negative control), treated with the vehicle (corn oil).
Group 3 (positive control), treated with the reference hypocholesterolemic drug atorvastatin, at a dose of 20 mg/kg b.w.
Group 4, treated with BCP at a dose of 30 mg/kg b.w. (BCP 30).
Group 5, treated with BCP at a dose of 100 mg/kg b.w. (BCP 100).
Group 6, treated with BCP at a dose of 300 mg/kg b.w. (BCP 300).

**Fig. 1:**
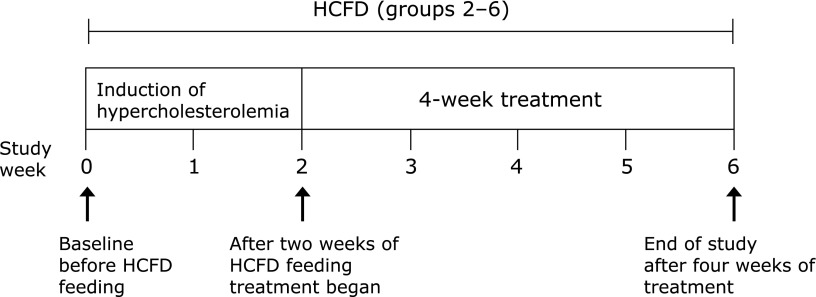
Study design: Except for group 1, all groups were subjected to high cholesterol and fat diet (HCFD) over a period of 6 weeks.

All treatments were given once a day in a volume of 0.5 ml/animal by oral gavage according to the protocol shown in Fig. 1. Doses of BCP were selected based on LD_50_ and previously published studies that indicated oral LD_50_ of BCP for rats to be >5,000 mg/kg.^(30–32)^

### Blood sampling

Blood was collected from the retrorbital plexus of the eye at 0, 2, 3, 4, 5 and 6 weeks. Animals were fasted overnight and lightly anaesthetized with diethyl ether. Two ml of blood was drawn, transferred to sterile vacutainers with gel, allowed to clot at room temperature for 1 h, centrifuged for 10 min at 3,000 rpm. Serum was separated and stored in Eppendorf tubes at −20°C for biochemical analyses. At the end of the experiment, rats were fasted overnight, weighed and sacrificed by an overdose of diethyl ether. More blood was collected (4 ml) through both eyes and serum was obtained and stored as mentioned above. Liver was removed, weighed, and a small part of the right lobe was excised, fixed in 10% formalin saline for histological examination.

### Measurement of lipid profile in serum samples

TC, TG and HDL levels were measured enzymatically using commercial assay kits according to the manufacturer’s instructions. LDL was calculated using the equations LDL = TC − [(HDL + very LDL (VLDL)], and VLDL = TG/5. The atherogenic index (AI) and HDL/TC ratio (HTR) were calculated for the last week of the experiment as follows:

$$AI=\frac{TC\;-HDL}{HDL}$$

$$HTR=\;\frac{HDL}{TC}\;\times 100$$

### Activity of hepatic marker enzymes in serum samples

Activities of AST, ALT, ALP and LDH were measured enzymatically in the serum sample for the last week of the experiment using commercial assay kits according to the manufacturer’s instructions.

### Activity of antioxidant enzymes in serum samples

#### Superoxide dismutase (SOD)

Serum SOD activity was measured on ELISA plate reader at a wavelength of 450 nm using a colorimetric assay kit according to the manufacturer’s instructions.

#### Catalase (CAT)

Serum CAT activity was measured on ELISA plate reader at a wavelength of 540 nm using a colorimetric assay kit according to the manufacturer’s instructions.

### Measurement of HMG-CoA reductase activity (HMG-CoA/mevalonate ratio) in liver homogenate

The activity of HMG-CoA reductase was measured in liver homogenate using the procedure of Rao and Ramakrishnan.^(33)^ The ratio of HMG-CoA to mevalonate was taken as an index of enzyme activity which catalyzes the conversion of HMG-CoA to mevalonate. Lower ratios indicate higher enzyme activity and *vice* *versa*.

### Histological examination of liver

Livers were removed from rats, fixed in 10% formalin saline, processed for paraffin embedding, cut into 5 µm-thick sections using rotary microtome, mounted on slides and stained with hematoxylin–eosin. Ten light microscopic fields of each section were examined and scored for the degree of steatosis under a compound light microscope at 200X. Liver steatosis was evaluated quantitatively as the percentage of hepatocytes containing macrovesicular fat using the grading and scoring system of Kawasaki *et al.*^(34)^ Macrovesicular fat refers to fat droplets similar to or larger than the size of the nucleus, often displacing the nucleus.^(35)^

### Statistical analysis

Data were presented as means ± SEM, and were analyzed using one-way analysis of variance (ANOVA) followed by post hoc Fisher’s least significant difference test. Differences were considered significant when *p*<0.05. Data were analyzed using GraphPad Prism software (ver. 7.03).

## Results

### Effect of BCP on lipid profile, AI, and HTR% of hypercholesterolemic rats

**Fig. 2:**
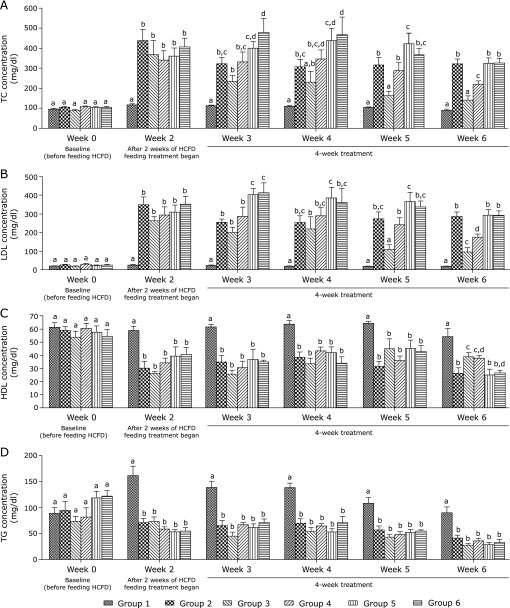
Effect of BCP on serum lipid profile of hypercholesterolemic rats. (A) TC concentration (mg/dl). (B) LDL concentration (mg/dl). (C) HDL concentration (mg/dl). (D) TG concentration (mg/dl). Group 1, normal + corn oil (vehicle) (*n* = 7); group 2, HCFD + corn oil (*n* = 8); group 3, HCFD + 20 mg/kg of atorvastatin (*n* = 7); group 4, HCFD + 30 mg/kg of BCP (*n* = 7); group 5, HCFD + 100 mg/kg of BCP (*n* = 6); group 6, HCFD + 300 mg/kg of BCP (*n* = 6). Data are expressed as means ± SEM and are analyzed using one-way ANOVA followed by Fisher’s least significant difference test. Different letters indicate significant differences at *p*<0.05 within each week for the same parameter.

Figure 2 shows that after two weeks of HCFD consumption, hypercholesterolemic rats (group 2) showed a significant increase in serum TC and LDL (*p*<0.0001 and *p*<0.0001, respectively) and a significant decrease in serum HDL and TG (*p*<0.0001 and *p*<0.0001, respectively) when compared to normal rats (group 1). These effects were detected from week 2 and continued throughout the duration of the experiment.

Treatment with 30 mg/kg of BCP (group 4) for four weeks caused a significant decrease in the serum TC as well as LDL (*p*<0.01 and *p*<0.001, respectively) compared to group 2. This decrease in TC and LDL in group 4 was smaller (*p*<0.05) than that in group 3 (atorvastatin-treated group) (Fig. 2A and B). Moreover, HDL level increased in group 4 and this increase was similar (*p*>0.05) to that observed in group 3 (Fig. 2C). Finally, this dose of BCP had no effect on serum TG. Serum TG started to decrease significantly (*p*<0.0001) in HCFD-fed rats in groups 2–6 starting from week 2 and continued through week 6 (Fig. 2D). In contrast, TC in group 5 which received 100 mg/kg of BCP was significantly higher (*p*<0.05) than that in groups 2 and 4 in week 5. Moreover, in week 3, LDL level in both groups 5 and 6 was significantly higher (*p*<0.01) in comparison with groups 2 and 4 (Fig. 2A and B). Also, TC in group 6 which received a high dose of BCP (300 mg/kg) was significantly higher (*p*<0.05) than that in group 2 during the first two weeks of treatment (weeks 3 and 4) and both TC and LDL increased significantly in groups 5 and 6 during week 6 when compared to group 4.

Table 1 shows that AI increased significantly (*p*<0.0001) while HTR% decreased significantly (*p*<0.0001) in group 2 when compared to group 1. However, treatment with BCP reversed these effects significantly, thus AI was reduced (*p*<0.001) and HTR% (*p*<0.05) was elevated in group 4 when compared to group 2. This reduction of AI was comparable (*p*>0.05) to that observed in group 3, while the elevation of HTR% was significantly less (*p*<0.01) than that of group 3. However, when comparing group 4 to groups 5 and 6, AI was significantly higher and HTR% significantly lower than those in group 4. Nevertheless, the decrease in TC, LDL and AI as well as the increase in HDL and HTR% induced by BCP were not detected in groups treated with higher doses (100 and 300 mg/kg) of BCP in comparison to group 2.

### Effect of BCP on the activity of HMG-CoA reductase of hypercholesterolemic rats

**Fig. 3:**
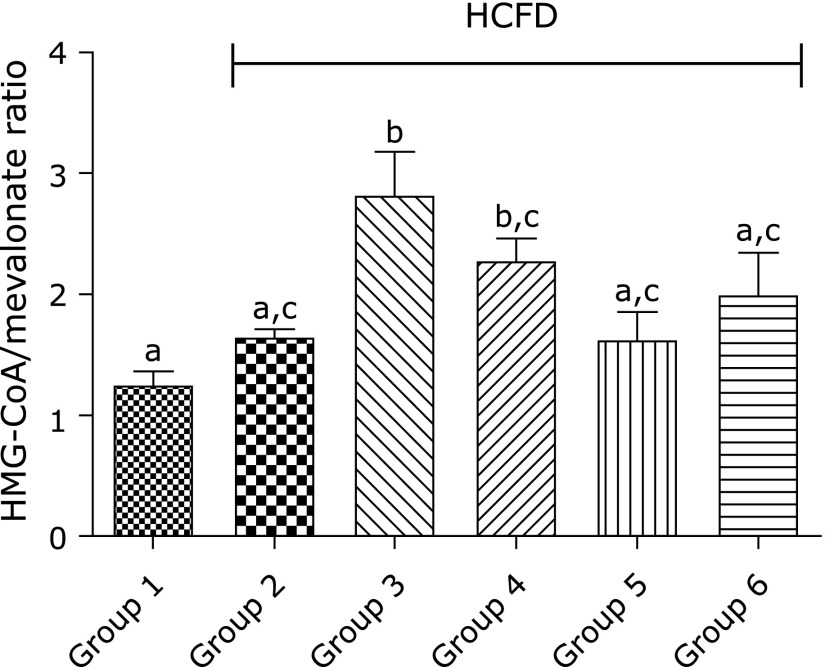
Effect of BCP on hepatic HMG-CoA/mevalonate by the end of the 6th week. Group 1, normal + corn oil (vehicle) (*n* = 7); group 2, HCFD + corn oil (*n* = 8); group 3, HCFD + 20 mg/kg of atorvastatin (*n* = 7); group 4, HCFD + 30 mg/kg of BCP (*n* = 7); group 5, HCFD + 100 mg/kg of BCP (*n* = 6); group 6, HCFD + 300 mg/kg of BCP (*n* = 6). Data are expressed as means ± SEM and are analyzed using one-way ANOVA followed by Fisher’s least significant differences test. Different letters indicate significant differences at *p*<0.05.

The ratio of HMG-CoA/mevalonate increased remarkably in group 3 as well as in group 4 compared to that in group 1 (*p*<0.001 and *p*<0.05, respectively), indicating a decrease in the activity of HMG-CoA reductase in groups 3 and 4. There was no significant difference (*p*>0.05) between group 3 and group 4 in the ratio of HMG-CoA/mevalonate (Fig. 3). This effect on HMG-CoA reductase was only observed with 30 mg/kg BCP but not with higher doses (100 and 300 mg/kg).

### Effects of BCP on weight, structure and function of the liver of hypercholesterolemic rats

**Fig. 4:**
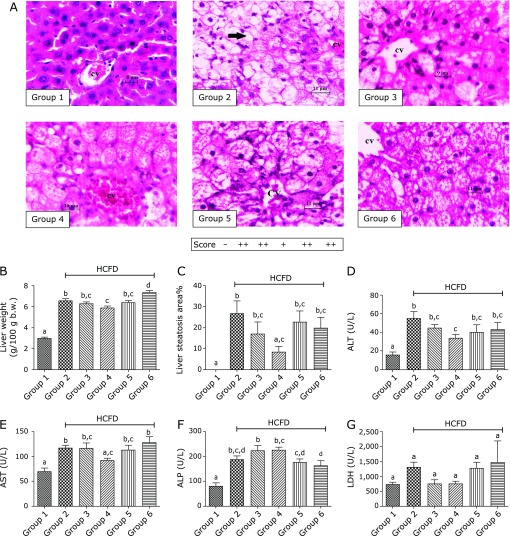
Effects of BCP on histological appearance of liver tissues, liver weight, steatosis (score and percentage) and hepatic marker enzymes by the end of week 6. (A) Liver section from rats in each of the groups stained with H&E, ×400. (B) Liver weight (g/100g b.w.). (C) Steatosis score and percentage. (D–G) Hepatic marker enzymes; (D) ALT, (E) AST, (F) ALP, (G) LDH. Group 1, normal + corn oil (vehicle) (*n* = 7); group 2, HCFD + corn oil (*n* = 8); group 3, HCFD + 20 mg/kg of atorvastatin (*n* = 7); group 4, HCFD + 30 mg/kg of BCP (*n* = 7); group 5, HCFD + 100 mg/kg of BCP (*n* = 6); group 6, HCFD + 300 mg/kg of BCP (*n* = 6). Note that the fatty changes comprising tiny and large vacuoles, pleomorphic nuclei and swelling hepatocytes are abundant in the negative control group (group 2), BCP 100 (group 5) and BCP 300 (group 6). Note also the partial recovery in atorvastatin-treated (group 3) and in BCP 30-treated group (group 4). Arrow indicated macrovesicular steatosis. CV, central vein. Data are expressed as means ± SEM and are analyzed using one-way ANOVA followed by Fisher’s least significant difference test Different letters indicate significant differences at *p*<0.05 for the same parameter.

Figure 4B shows that hypercholesterolemic rats (group 2) exhibited liver enlargement (*p*<0.0001) as compared to normal rats (group 1). Comparatively, group 4 which was treated with 30 mg/kg BCP showed less increase (*p*<0.01) in liver weight as compared to group 2.

Histological examination of rat livers demonstrated that the morphology of hepatocytes from group 2 has changed when compared to normal hepatocytes from group 1; the cells became larger in size, filled with fat droplets, and the nucleus was displaced from the center. In contrast, lipid accumulation was dramatically less in livers of group 4 compared to those of group 2 where the individual cells appeared to be filled completely with lipid (Fig. 4A). As shown in Fig. 4C, the percentage of hepatic steatosis was significantly reduced (*p*<0.05) in group 4 as compared to that in group 2. This effect was not as obvious in group 5 or group 6.

The activity of serum ALT, AST and ALP was markedly elevated (*p*<0.0001, *p*<0.001 and *p*<0.0001, respectively) in group 2 compared to group 1. Treatment of group 4 with 30 mg/kg BCP caused a significant decrease in serum ALT and AST activities (*p*<0.05) but not ALP and LDH (*p*>0.05) as compared to group 2 (Fig. 4D–G). These effects of BCP on liver enzymes of hypercholesterolemic rats could not be observed in rats treated with higher doses (100 and 300 mg/kg) of BCP (groups 5 and 6). In fact, AST activity was increased in group 6 when compared to group 4.

### Effect of BCP on the antioxidant status

**Fig. 5:**
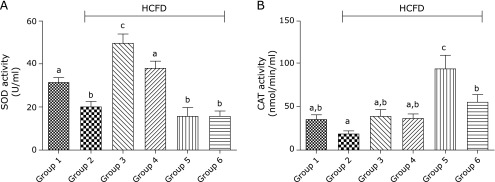
Effect of BCP on the activity of antioxidant enzymes by the end of the 6th week. (A) SOD activity (U/ml). (B) CAT activity (nmol/min/ml). Group 1, normal + corn oil (vehicle) (n = 6); group 2, HCFD + corn oil (*n* = 8); group 3, HCFD + 20 mg/kg of atorvastatin (*n* = 7); group 4, HCFD + 30 mg/kg of BCP (*n* = 7); group 5, HCFD + 100 mg/kg of BCP (*n* = 6); group 6, HCFD + 300 mg/kg of BCP (*n* = 5). Data are expressed as means ± SEM and are analyzed using one-way ANOVA followed by Fisher’s least significant difference test. Different letters indicate significant differences at *p*<0.05 for the same parameter.

The activity of serum SOD was suppressed (*p*<0.05) in group 2 compared to that in group 1. In contrast, the activity of serum SOD was elevated (*p*<0.001) in group 4 when compared to group 2 (Fig. 5A). No significant difference was observed in the activity of this enzyme between group 1 and group 4. In groups 5 and 6, the activity of CAT, but not those of SOD, increased significantly (*p*<0.0001 and *p*<0.01, respectively) in comparison to group 2 (Fig. 5B). The activity of SOD rather significantly decreased in groups 5 and 6 when compared to group 4.

## Discussion

HCFD used in this study was very effective in inducing hypercholesterolemia in Wistar rats after two weeks of feeding. This diet caused a significant elevation in serum TC and LDL and a significant reduction in serum HDL and TG in rats of group 2 in comparison with rats fed the normal diet (group 1). These effects were maintained from week 2 till the end of the experiment. The decrease in TG level in HCFD-fed groups compared to those fed the normal diet (Fig. 2D) could be due to lowering the carbohydrate content by 25% in HCFD compared to the normal diet. Feldman and co-workers showed that carbohydrate intake is the key factor influencing serum TG and VLDL.^(36)^ This effect results from the finding that feeding a low carbohydrate enhances TG clearance from circulation and reduces hepatic TG production via less VLDL synthesis and secretion in the circulation.^(37)^

In the present work, the daily administration of 30 mg/kg of BCP to hypercholesterolemic rats for four successive weeks caused an obvious decrease in serum TC as well as LDL (31.6% and 39.1%, respectively), although this drop in TC and LDL was smaller than that induced by the reference hypocholesterolemic drug atorvastatin (56.6% and 66.8%, respectively). Additionally, this dose of BCP increased HDL level by 43.4%, an effect that is comparable to that induced by atorvastatin (47.1%). Nevertheless, this dose of BCP had no detectable effect on TG level, presumably due to low carbohydrates portion in the diet as discussed above. These observations are consistent, in part, with the findings of a recent study using Triton WR-1339 induced hyperlipidemia in rats.^(38)^ This study found that BCP reduced total cholesterol, triglycerides and LDL cholesterol, did not increase HDL cholesterol, inhibited HMG-CoA reductase activity and ameliorated the antioxidant system.

AI and HTR% have been considered as indicators for atherogenesis.^(39)^ In the present experiments, 30 mg/kg of BCP suppressed the high level of AI to a degree comparable to that induced by atorvastatin and it elevated the low level of HTR% in hypercholesterolemic rats.

Interestingly, BCP seemed to have inhibited HMG-CoA reductase, the rate-limiting enzyme in cholesterol biosynthetic pathway, and this inhibition was comparable to that induced by atorvastatin. This observation indicates that the hypocholesterolemic effect of 30 mg/kg of BCP is due to inhibition of endogenous cholesterol synthesis.

Liver is generally considered as the primary organ responsible for maintaining cholesterol homeostasis. In the current study, hypercholesterolemic rats showed enlarged liver with pale coloration and fragile texture as compared to normal rats. Hepatomegaly can be attributed to the high fat content.^(40)^ It is believed that high serum cholesterol level damages hepatocytes leading to malfunctioning of the liver, through macro- and microvesicular steatosis.^(41)^ Histological examination of hypercholesterolemic rat livers, demonstrated the accumulation of lipid (moderate macrosteatosis), loss of hepatocytes integrity, hepatocytes enlargement and displacement of nucleus.

On the other hand, HCFD-fed rats treated with 30 mg/kg of BCP showed less liver enlargement, suggesting less lipid deposition in hepatocytes. Histological examination showed that lipid accumulation in hepatic cells from animals treated with 30 mg/kg of BCP decreased, indicating that the oral administration of 30 mg/kg of BCP could relieve liver stress caused by hypercholesterolemia and attenuate the severity of fatty liver. Indeed, the percentage of hepatic steatosis supported these observations, since steatosis was significantly reduced by the treatment with 30 mg/kg of BCP. In support of this, Calleja *et al.*^(16)^ reported that BCP reduced the degree of steatosis and the extent of liver fibrosis induced by carbon tetrachloride (CCl_4_) in rats. Recently, Kamikubo and his coworkers also demonstrated the role of BCP in inhibiting lipid accumulation in hepatocyte at the molecular level.^(24)^ They showed that BCP stimulates the activation of AMPK by phosphorylation, an effect mediated by CB2 receptor-dependent Ca^2+^ signaling pathway. The activated AMPK signaling pathway enhances fatty acid oxidation and attenuates lipogenesis.

In the present work, 30 mg/kg of BCP reduced serum ALT and AST effectively, which are known to be marker enzymes for liver injury, an observation that indicates that BCP protects against liver injury induced by HCFD and improves liver functions. Similar observations were reported by Calleja *et al.*^(16)^ where BCP suppressed the elevated activities of ALT, AST, ALP and LDH in CCl_4_-treated rats. In another study, Shyamala and coworkers found that cloves could decrease serum ALT and AST in hyperlipidemic rats, an effect which may be due, in part, to BCP as one of its main bioactive components.^(42)^

It has been shown that hypercholesterolemia minimizes the effectiveness of the antioxidant defense system by reducing the activity of CAT and SOD in rats.^(5)^ Our data supports this observation and it also showed that 30 mg of BCP reversed this effect by elevating the antioxidant enzyme activity of serum SOD (and CAT although it didn’t reach a statistical significance) to a level close to that observed in normal rats, suggesting that BCP has boosted the antioxidant systems. This observation is consistent with a recent study which showed that oral administration of BCP to diabetic rats improved the activities of SOD and CAT.^(31)^ Similarly, Calleja *et al.*^(16)^ reported that BCP was an effective inhibitor of lipid peroxidation, probably due to its free radical-scavenging activity against hydroxyl radicals, superoxide anions, and lipid peroxides. Moreover, it has been found that clove, a plant rich in BCP, could increase the activity of SOD and CAT in hyperlipidemic rats.^(42)^ The antioxidant capacity of BCP may be related to its chemical structure such as the presence of a bicyclic system with double rings.^(17)^

Although higher doses of BCP (100 and 300 mg/kg) seemed to increase the levels of TC and LDL at the beginning of the treatment period, they had no significant effect on these parameters at the end of the experiment when compared to group 2. Furthermore, these higher doses of BCP did not ameliorate the studied deleterious effects caused by HCFD. A possible explanation for that could be the toxicity associated with high doses of BCP, as manifested by the evident hepatomegaly in the group treated with 300 mg/kg of BCP (Fig. 4B). This suggestion is supported by the reported adverse effects of BCP such as bile duct disorder, hepatotoxicity, liver necrosis and liver weight gain.^(43)^ The selection of such high doses in our study was built on observations from literature suggesting that higher doses of BCP have beneficial effects when used under different experimental settings. For example, Cho *et al.*^(30)^ found that oral administration of 300 mg/kg BCP for 7 days suppressed colitis effectively in mice. Also, Calleja *et al.*^(16)^ found that oral pretreatment with 2, 20 and 200 mg/kg BCP protected against liver damage caused by CCl_4_ in a dose-dependent manner in rats. Furthermore, Basha and Sankaranarayanan used BCP orally in a dose of 200 mg/kg in streptozotocin-induced diabetic rats for 45 days and found that BCP ameliorated deranged glycoprotein metabolism, decreased hyperglycemia, and mitigated oxidative/inflammatory stress.^(31,44)^ The discrepancy between our data and these studies could be due to the fact that we applied the treatment for a relatively longer duration. In nature, the active ingredients are found in medicinal plants in little amounts; therefore, using the active ingredient in a pure form and in large amounts may give undesirable effects.

In conclusion, BCP plays an effective role in lowering TC and LDL levels, increases HDL level, and reduces hepatic lipid accumulation in hypercholesterolemic rats when used in a dose of 30 mg/kg. The underlying mechanism of cholesterol lowering action is likely to be through scavenging ROS, leading to deactivation of HMG-CoA reductase and inhibition of endogenous cholesterol synthesis. These observations indicate that BCP may be further examined as a useful therapeutic drug in treating hypercholesterolemia and fatty liver disease.

## Figures and Tables

**Table 1:** Effect of BCP on atherogenic index (AI) and ratio of HDL to total cholesterol (HTR%)

Parameters	Group 1	Group 2	Group 3	Group 4	Group 5	Group 6
AI	0.70 ± 0.11^a^	13.56 ± 2.53^b^	2.89 ± 0.91^a^	4.92 ± 0.63^a^	13.40 ± 2.92^b^	11.66 ± 1.35^b^
HTR%	60.19 ± 3.69^a^	8.48 ± 1.42^b^	32.32 ± 5.08^c^	18.16 ± 2.07^d^	8.12 ± 2.19^b^	8.44 ± 1.03^b^

Atherogenic index and HDL/TC ratio were calculated for rats by the end of the last week of the experiment. Group 1, normal + corn oil (vehicle) (*n* = 7); group 2, HCFD + corn oil (*n* = 8); group 3, HCFD + 20 mg/kg of atorvastatin (*n* = 7); group 4, HCFD + 30 mg/kg of BCP (*n* = 7); group 5, HCFD + 100 mg/kg of BCP (*n* = 6); group 6, HCFD + 300 mg/kg of BCP (*n* = 6). Data are expressed as means ± SEM and are analyzed using one-way ANOVA followed by Fisher’s least significant difference test. Different letters in the same row indicate significant differences at *p*<0.05.

## Abbreviations

AI: atherogenic index
ALP: alkaline phosphatase
ALT: alanine aminotransferase
AMP: 2-amino-2-methyl-1-propanol
AMPK: AMP-activated protein kinase
AST: aspartate aminotransferase
b.w.: body weight
BCP: β-caryophyllene
CAT: catalase
CB2: cannabinoid-2
CCl_4_: carbon tetrachloride
ELISA: enzyme linked immunosorbent assay
HCFD: high cholesterol and fat diet
HDL: high density lipoprotein
HMG-CoA: hydroxy-methylglutaryl coenzyme A
HTR: high density lipoprotein to total cholesterol ratio
LDH: lactate dehydrogenase
LDL: low density lipoprotein
ROS: reactive oxygen species
SOD: superoxide dismutase
TC: total cholesterol
TG: triglyceride
VLDL: very low density lipoprotein
